# Clinical risk predictors associated with cardiac mortality following vascular surgery in South African patients

**Published:** 2007

**Authors:** BM Biccard, R Bandu

**Affiliations:** Nelson R Mandela School of Medicine, Durban Nuffield Department of Anaesthetics, University of Oxford, John Radcliffe Hospital, Oxford, UK; Department of Clinical Technology, Durban University of Technology, Durban

## Abstract

Clinical risk prediction is important in the prognostication of peri-operative cardiac complications and the management of high-risk cardiac patients for major non-cardiac surgery. However, the current pre-operative clinical risk indices have been derived in European and American patients and not validated in South African patients.

The purpose of this study was to evaluate the utility of the clinical risk predictors identified in L ee’s revised cardiac risk index and in the African arm of the INTERHEART study, in predicting cardiac mortality following vascular surgery in South African patients.

A retrospective cohort study was conducted of all patients undergoing elective or urgent vascular surgery at Inkosi Albert L uthuli Central Hospital over a three-year period. All in-hospital deaths were identified and classified into cardiac or non-cardiac deaths by an investigator blinded to the patients’ pre-operative clinical risk predicators. A second investigator blinded to the cause of death identified the following clinical risk predictors: history of ischaemic heart disease, congestive cardiac failure and cerebrovascular accident, presence of diabetes, hypertension and obesity (BMI > 30 kg.m^–2^), elevated serum creatinine (> 180 μmol. l^–1^), positive smoking history and ethnicity.

The main finding was that a serum creatinine level of greater than 180 μmol.l^-1^ and a positive smoking history were significantly associated with cardiac death (*p* = 0.012, *p* = 0.012, respectively). Multivariate analyses using a backward stepwise modeling technique found only a serum creatinine of > 180 μmol.l–1 and a positive smoking history to be significantly associated with cardiac mortality (*p* = 0.038, 0.035, respectively) with an odds ratio and 95% confidence interval of 3.02 (1.06−8.59) and 3.40 (1.09−10.62), respectively. All other clinical predictors were not significantly different between the two groups.

However, based on the sample size of this study, a type 2 or b error may have resulted in the other risk predictors not being identified as important clinical predictors of cardiac mortality. Therefore, until such time as a study of adequate power is conducted, a history of ischaemic heart disease, congestive cardiac failure, diabetes and cerebrovascular accidents should still be considered to be important clinical risk predictors in South African surgical patients.

In conclusion, an elevated serum creatinine and a positive history for smoking are important clinical predictors of cardiac mortality in South African patients following elective or urgent vascular surgery.

## Summary

It is estimated that annually between 500 000 and 900 000 patients sustain major cardiac complications following non-cardiac surgery worldwide.^1^ In order to decrease major perioperative cardiac complications, a Bayesian approach was applied to the pre-operative work-up.^2^ Integral to determining the applicability of pre-operative cardiac investigation is the prognostication of major cardiac complications, based on the presence of clinical risk predictors. This has led to clinical risk indices in order to quantify peri-operative cardiac risk.^3^-^5^

Although the first two (Goldman’s and Detsky’s) risk indices were not significantly different in prognostic ability,^6^ the more recently published revised cardiac risk index^5^ has been found to be significantly better at predicting peri-operative cardiovascular complications^5^ and it has also been validated outside of its derivation population.^7^ While recent cardiac risk indices have been shown to have similar predictive values for the derivation and validation cohorts,^5^,^8^ this is not surprising as the populations from which these risk indices were derived and then validated were the same.

For these risk indices to be applicable in South Africa they should fulfil two criteria. Firstly, South Africans patients should be of a similar cardiac risk to the North American and European patients where these indices were derived. Secondly, the relative importance or ‘weighting’ of the clinical risk predictors associated with peri-operative cardiac complications needs to be similar to that reported in the international literature. Unfortunately neither of these criteria is met, and the applicability of these cardiac risk indices in South Africa is therefore contentious.

It now appears that South Africans are at higher cardiac risk than North American and European populations. A process of epidemiological transition has been described to illustrate the progression of cardiovascular disease in a population.^9^ Exposure to a ‘western lifestyle’ is integral to increasing cardiovascular risk. This is prevalent in South African society today. However, South Africans are considered to be at higher cardiovascular risk than individuals from more developed countries, as we do not have similar levels of monitoring and managing this cardiovascular disease.^9^ One would therefore expect the cardiac risk associated with the European and American clinical risk indices to be lower than that found in the South African population. 5,8 This process of epidemiological transition would also suggest that within South Africa, the various population groups comprising our population have potentially different degrees of cardiovascular risk, due to differences in socio-economic and educational status.^10^

It also appears that the risk factors associated with myocardial infarction in South Africans do not necessarily carry the same weight as that of the North American and European populations. Five risk factors in the INTERHEART study were shown to be associated with first-time acute myocardial infarction (AMI) in sub-Saharan Africans. These include a history of smoking, diabetes, hypertension, abdominal obesity and the ratio of apolipoprotein B to apolipoprotein A-1.^10^ These five risk factors could account for 89.2% of the risk associated with AMI.^10^

Hypertension and diabetes were significantly more important risk factors for AMI in the African INTERHEART study in comparison with the global INTERHEART study. Abdominal obesity is also a significantly stronger risk factor in the African INTERHEART study than the global study. Smoking status and permanent stress are also significant predictors of AMI in the African group. Indeed as the odds ratio (OR) of a number of these risk factors is higher than that of the overall INTERHEART study, the cardiovascular burden in Africa may well be larger, due to uncontrolled/undiagnosed/poorly managed major cardiovascular risk factors,^10^ (which is what we would expect from our understanding of epidemiological transition).^9^

The variance of risk factors associated with myocardial infarction between sub-Saharan Africans and the globally accepted risk factors,^10^ and the inter-ethnic differences in risk factors associated with acute coronary syndromes in South Africans^10^-^12^ suggest that the clinical risk indices of peri-operative cardiovascular risk that are not derived in South Africa may be of limited value when applied to South African patients.

The aim of this study was therefore to evaluate whether the clinical risk predictors identified in the revised cardiac risk index5 and in the INTERHEART study^10^ were associated with cardiac mortality in South African patients who underwent elective or urgent vascular surgery.

## Methods

Ethics approval was granted by the Ethics committee of the Nelson R Mandela School of Medicine for this study. A retrospective cohort study was conducted using the computerised hospital information system at Inkosi Albert Luthuli Central Hospital. All elective or urgent vascular surgical procedures between 1 June 2003 and 1 June 2006 were identified. Urgent surgery was defined as surgery scheduled for the next elective list. All patients who suffered in-hospital deaths following these procedures were identified.

From the hospital database the presence of the following risk factors were identified (by RB): history of ischaemic heart disease (or pathological Q waves on ECG), congestive cardiac failure and cerebrovascular accident, presence of diabetes, serum creatinine level of greater than 180 μmol.l^–1^, age, gender, hypertension, body mass index above 30 kg.m^–2^, history of smoking, and ethnicity. RB was blinded to the cause of death. BB assigned the cause of death as cardiac or non-cardiac; he was blinded to the risk factors of the patients.

Cardiac deaths were defined as a postoperative cardiac event being the primary event that subsequently resulted in death. These cardiac events included cardiac arrest, myocardial infarction or cardiac failure. A primary cardiac arrest was defined as a witnessed cardiac arrest associated with ventricular fibrillation, ventricular tachycardia or asystole in a patient who was previously considered stable. Myocardial infarction was defined as postoperative clinical signs or symptoms consistent with myocardial ischaemia and either an associated diagnostic rise in troponin T or creatinine kinase MB, or electrocardiographic (ECG) changes consistent with acute myocardial infarction. Primary postoperative cardiac failure was defined as clinical signs and symptoms consistent with acute pulmonary oedema requiring inotropic support without an obvious precipitant.

In order to identify these cardiac events, BB assessed postoperative clinical notes, drug prescriptions, nursing observations and postoperative investigations. These included evaluation of laboratory investigations (creatine kinase MB fraction, troponin T), radiographic (chest X-ray features consistent with congestive heart failure) and new changes on ECG, when compared to the pre-operative ECG. The patients assigned to the non-cardiac death group would be the control group for the patients who died primarily of cardiac causes.

## Statistical analyses

All categorical data were analysed using descriptive statistics and either the Fisher’s exact test or Pearson’s chi-square test where appropriate. All continuous data were analysed using descriptive statistics and compared using independent samples *t*-test. Multivariate analysis was conducted using binary logistic regression analysis. Clinical risk factors were entered into the model. A backward stepwise modeling technique was used, based on likelihood ratios with entry and removal probabilities set at 0.05 and 0.1, respectively. The interaction between significant risk factors was also tested. The odds ratio for cardiac death and 95% confidence intervals (CI) are reported. SPSS 13.0 for Windows (1 Sept 2004) was used for data analyses.

## Results

A total of 1 360 vascular procedures were identified for the three-year period. There were 83 deaths following these elective or urgent vascular surgical procedures, comprising 24 cardiac deaths and 59 non-cardiac deaths [54 (65%) males and 29 (35%) females]. The mean age (± 1 SD) was 62.1 (± 12.3) years, with no statistically significant difference in age between the groups (*p* = 0.439).

The primary precipitant of all the peri-operative deaths is shown in Table 1. The presence of the clinical risk factors, the cause of death and the statistical analyses are shown in Table 2.. Cardiac failure at the time of surgery was well controlled in the two patients who presented with a history of congestive cardiac failure.

**Table 1:** The Primary Cause Of Perioperative Death

*Primary cause of death*	*Number (%)*
Cardiac deaths	
Myocardial infarction	16/24 (67)
Cardiac failure	3/24 (12)
Cardiac arrest	5/24 (21)
Non-cardiac deaths	
Massive haemorrhage	10/59 (17)
Acute renal failure	5/59 (8)
Cerebrovascular accident	8/59 (14)
Respiratory failure	6/59 (10)
Sepsis	7/59 (11)
Abdominal compartment syndrome	1/59 (2)
Hypoglycaemia	1/59 (2)
Primary cause indeterminate	21/59 (36)

**Table 2:** Clinical Risk Factors And Associated Mortality Following Vascular Surgery

*Clinical risk factors*	*Cardiac death (%)*	*Non-cardiac death (%)*	p*-value*
Male gender	18/24 (75)	36/59 (61)	0.226*
Ischaemic heart disease	15/24 (63)	24/59 (41)	0.071*
Congestive cardiac failure	1/24 (4)	1/59 (2)	0.497^†^
Cerebrovascular accident	2/24 (8)	7/59 (12)	1.00^†^
Diabetes	8/24 (33)	24/59 (41)	0.533*
Hypertension	17/27 (71)	39/59 (66)	0.677*
BMI > 30 kg.m^–2^	2/24 (8)	5/59 (9)	1.00^†^
Creatinine > 180 μmol.l^–1^	12/24 (50)	13/59 (22)	0.01*
Smoker	19/24 (79)	29/59 (49)	0.01*

*Pearson chi-square test, ^†^Fisher’s exact test.

The mortality associated with the various ethnic groups is shown in Table 3. The ethnicity of seven patients was unknown.

**Table 3:** Ethnicity And Associated Mortality Following Vascular Surgery

*Ethnicity*	*Cardiac death (%)*	*Non-cardiac death (%)*	p*-value*
Asian	15 (34)	29 (66)	0.68*
Black African	2 (11)	17 (89)	
White European	6 (46)	7 (54)	

*Pearson chi-square test.

The risk factors included in the logistic regression were elevated serum creatinine, smoking, gender, a history of ischaemic heart disease, diabetes and ethnicity. Only an elevated creatinine and a smoking history were not excluded. The odds ratio (95% CI) for cardiac death associated with a creatinine level > 180 μmol.l^–1^ was 3.02 (1.06−8.59) and for a history of smoking was 3.40 (1.09−10.62) with *p*-values of 0.038 and 0.035, respectively. There was no interaction between these two clinical risk factors.

## Discussion

The prevalence of cardiac clinical risk predictors in our vascular patients was similar to or higher than that reported in European vascular patients^8^ (Table 4), with the exception of congestive cardiac failure. This was consistent with the INTERHEART study^10^ and epidemiological transition of cardiovascular disease in sub-Saharan Africa.^9^

**Table 4:** Prevalence Of Cardiac Clinical Predictors In South African And Dutch Vascular Patients

*Clinical risk factor*	*South African patients (%)*	*Dutch patients (%)*
Ischaemic heart disease	41	≤ 40
Congestive cardiac failure	2	3.8
Cerebrovascular accident	12	13.5
Diabetes	41	11
Serum creatinine > 180 μmol.lM^-1^	22	3.6

The two patient groups in this study can be considered comparable. We controlled for surgical risk by only analysing vascular surgical patients who were known to be at the highest risk for major postoperative cardiac complications.^5^ Similarly, the patients in both groups were found to be of a comparable age, which was important, as increasing age is a risk factor for postoperative cardiac morbidity.^5^

This study suggests that a serum creatinine level of greater than 180 μmol.l^–1^ and a history of smoking are important clinical risk predictors of cardiac mortality in high-risk South African vascular surgical patients. An elevated serum creatinine was found to have the highest relative risk of major cardiac complications following non-cardiac surgery in the derivation cohort of the revised cardiac risk index, with an OR (95% CI) of 3.0 (1.4−6.8),^5^ which was similar to our finding. Validation of this risk index in a Dutch population found an elevated creatinine to be the second most important clinical predictor of major cardiac complications after a history of cardiac failure.^9^

The finding that a history of smoking increased the risk for cardiac death following vascular surgery three-fold was important. Surprisingly, a history of smoking was not evaluated as a clinical predictor in the pre-operative cardiac risk indices.^3^-^5^, ^8^ A positive smoking history has not been associated with all-cause mortality following non-cardiac surgery in an American veteran affairs hospital study.^13^ A history of smoking was, however, a significant risk factor associated with AMI in non-surgical South African patients.^10^ Besides being an important clinical predictor in our South African vascular patients, this finding suggests that as a clinical risk predictor, smoking should be further evaluated in pre-operative cardiac risk prediction for non-cardiac surgery in other centres.

Although, all the other clinical risk predictors were found to be non-significant determinants of peri-operative cardiac mortality, our study did not have the power to determine if these factors were important clinical factors based on the odds ratios for major cardiovascular complications published in the derivation cohort of Lee’s revised cardiac risk index.^5^ Based on two controls per case and an OR for major cardiovascular complications associated with a history of ischaemic heart disease, congestive cardiac failure, diabetes and cerebrovascular accidents of 2.4, 1.9, 3.0 and 3.2, respectively,^5^ the sample size we needed for a significance of 0.05 and a power of 80% was 187, 454, 120 and 195 patients, respectively.^14^ This study was, therefore, underpowered to determine whether a history of ischaemic heart disease, cerebrovascular accidents, congestive cardiac failure or diabetes are important clinical predictors of peri-operative cardiac death in South African patients.

However, the trends identified in our study were also consistent with known clinical predictors of peri-operative cardiacmorbidity.^3^-^5^, ^8^ Male gender, a history of ischaemic heart disease and congestive cardiac failure were all more common in the patients who suffered a cardiac death. Male gender was associated with increased major cardiac complications following non-cardiac surgery.^5^,^8^ A history of ischaemic heart disease has consistently been associated with increased peri-operative cardiac risk.^3^-^5^,^8^ Congestive cardiac failure has consistently been shown to be one of the most important predictors of major cardiac morbidity following non-cardiac surgery.^3^-^5^,^8^

Only two patients in our cohort were found to have a history of congestive cardiac failure, and one of these patients died. The prevalence of heart failure in our population presenting for elective or urgent vascular surgery was also lower than expected.^15^ It is likely, however, that cardiac failure is an important clinical predictor of cardiac risk in South African vascular patients.^15^

Although diabetes and hypertension were the most important clinical risk factors associated with AMI in the African arm of the INTERHEART study,^10^ these risk factors were not consistently associated with an adverse cardiac outcome in the peri-operative literature.^5^,^8^ Diabetes was not significantly associated with cardiac mortality in the only risk index of vascular surgical patients^8^ and it was not significantly associated with major cardiovascular complications in the validation cohort of the revised cardiac risk index.^5^

Hypertension, similarly, was non-predictive of an adverse cardiac outcome. A meta-analysis of hypertension in perioperative patients suggests that it was statistically associated with cardiac morbidity, although the clinical importance of this finding was more difficult to quantify.^16^ It is possible that hypertension in the presence of a previous cerebrovascular accident may be an important clinical risk predictor, particularly in black South African patients.^17^ Unfortunately our study did not have the power to address this issue. This is an area that should receive special attention in future South African epidemiological studies.

Although obesity is a risk factor for AMI,^10^ it was not found as a cardiac risk predictor in the peri-operative risk indices.^3^-^5^,^8^ Again our numbers were probably too small to evaluate this risk factor. Obesity has, however, not been predictive of all-cause mortality in a veteran affair’s study.^13^

Our study had a number of limitations. Firstly, it was a retrospective study, and therefore it is possible that not all the risk predictors were recorded in the pre-operative medical charts. Similarly, it is possible that unavailable postoperative investigations may have resulted in the incorrect classification of the primary cause of death. Secondly, the identification of patients considered to be at high risk for peri-operative cardiac complications may have resulted in bias with altered peri-operative medical management^18^,^19^ and therefore a better peri-operative outcome in the high-risk group.^20^ Thirdly, this study was underpowered to assess the importance of ischaemic heart disease, congestive cardiac failure, diabetes and a history of cerebrovascular accidents in predicting peri-operative cardiac death. It is possible that all these factors are important in South Africans and until such a study is published, they should all be considered important prognostic markers. Finally, this was a study of only vascular surgical patients. Hence, this study cannot be extrapolated to patients undergoing other types of surgery, especially as vascular surgery is associated with the highest risk for major peri-operative cardiac complications.^5^

However, despite these study limitations, it appears that a serum creatinine level greater than 180 μmol.l^–1^ and a history of smoking are important clinical risk predictors of cardiac mortality in high-risk South African vascular surgical patients. These findings suggest that the relative importance or ‘weighting’ of clinical risk predictors for cardiac mortality are not necessarily the same in South African patients, when compared with European and American patients, as smoking has not been shown to be an independent predictor of cardiac mortality in the European and American literature.

It is therefore imperative that we continue to identify clinical predictors in South African patients, as it is likely that they are different from that published in the international literature. Identification of clinical risk predictors in our population will allow for rational peri-operative medical management,^18^,^19^ and pre-operative investigation.^02^
